# Paratesticular metastasis from colorectal adenocarcinoma presenting as hydrocele: a rare case report and literature review

**DOI:** 10.3389/fonc.2024.1373760

**Published:** 2024-04-05

**Authors:** XiaoJun Huang, KeLi Xu, Yin Zhao, MinHui Chen, ZheYang Li

**Affiliations:** ^1^ Department of Urology, The Second Affiliated Hospital Zhejiang University School of Medicine, Hangzhou, Zhejiang, China; ^2^ The Second school of Clinical Medicine, Second Clinical Medical College of Zhejiang Chinese Medical University, Hangzhou, Zhejiang, China

**Keywords:** colorectal neoplasm, testicular neoplasm, paratesticular metastasis, testicular hydrocele, neoplasm metastasis, therapeutics, case report

## Abstract

Colorectal cancer, with the liver being the most common site of distant metastasis, followed by the lungs and bones. Although reports of metastasis to the testis exist, paratesticular metastasis is extremely rare. A 37-year-old male presented with scrotal swelling. Ultrasound revealed hydrocele of the tunica vaginalis. The patient underwent routine surgical treatment, and postoperative pathology of the tunica vaginalis indicated adenocarcinoma of gastrointestinal origin. Colonoscopic biopsy confirmed adenocarcinoma of the sigmoid colon. After six months of systemic therapy, tumor reduction surgery was performed in conjunction with tunica vaginalis excision. Postoperative pathology suggested histological similarity in both sites, with immunohistochemistry results supporting the diagnosis of sigmoid colon adenocarcinoma metastasizing to the tunica vaginalis. We conducted a literature review, summarizing and discussing clinical presentations, metastatic pathways, and diagnostic approaches.

## Introduction

Hydrocele of the tunica vaginalis is a common urological condition with various etiologies. Despite its rarity, the impact of tumor-related hydrocele cannot be overlooked, as it significantly influences the overall diagnostic and therapeutic approach to the disease. Both testicular tumors and adjacent tumors can lead to hydrocele. In recent years, the incidence of colorectal cancer has been steadily rising in China, accompanied by an increase in mortality rates (1). Common sites of metastasis for colorectal cancer include the liver, lungs, and bones, while rare sites involve the testicles and adjacent tissues (spermatic cord, tunica vaginalis, etc.). We conducted a literature search on PubMed regarding cases of colorectal cancer metastasizing to the adjacent testicular tissues and identified several relevant reports. This phenomenon is predominantly observed in elderly males, with mucinous adenocarcinoma being the primary histological type (2–7). Herein, we present a case involving a young male patient who presented with scrotal effusion as the initial symptom. Postoperative pathology revealed colorectal adenocarcinoma with metastasis to the testicular tunica vaginalis. We provide a detailed description of the diagnostic and therapeutic processes, and discuss the relevant aspects of symptom presentation, pathological type, metastatic pathways, and treatment modalities in this particular case.

## Case report

**Figure 1:**
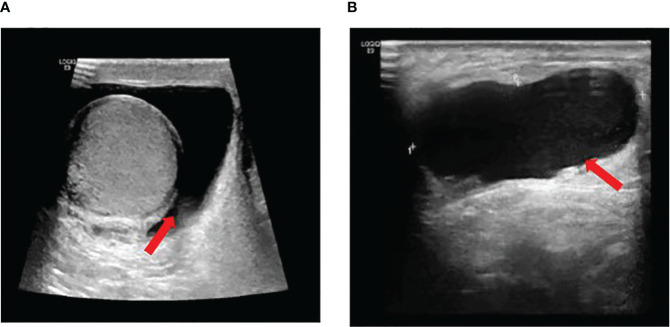
**(A)** Normal size of the right testicle, regular morphology, clear contour, smooth envelope, no apparent focal lesions in the parenchyma, and scattered free fluid hypoechoic areas visible around the testicle. **(B)** Visible patchy free fluid hypoechoic area around the testicle and spermatic cord, measuring approximately 4.31*1.48 cm, with good through-transmission internally.

A 37-year-old male with no underlying diseases presented with scrotal swelling for several months. He reported no significant discomfort in the medical history. Physical examination revealed positive transillumination test, and no palpable mass was detected, suggesting a possibility of tunica vaginalis fluid accumulation. Subsequent ultrasonography confirmed the diagnosis of testicular tunica vaginalis hydrocele, with no apparent occupancies in the testis, epididymis, or spermatic cord (**Figure 1**).

**Figure 2:**
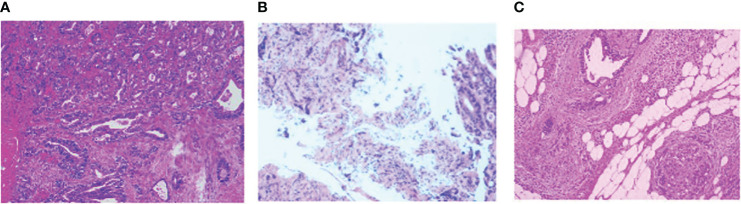
**(A)** Left testicular tunica albuginea fibrous capsule-like tissue with occasional atypical glandular structures in the interstitium, suggestive of a tumor; Right testicular tunica albuginea and appendage tissue reveal atypical glandular structures, indicating a possibility of metastatic adenoma, considering the immunohistochemical results, likely of gastrointestinal origin; **(B)** tissue biopsy confirmed adenocarcinoma of the sigmoid colon; **(C)** Colon adenocarcinoma with differentiation in the transverse colon, post-chemotherapy, measuring approximately 2.1*1cm in size.The Immunohistochemical results is same: CK7-,CK20+,PSA-,MLH1+,MSH2+,MSH6+,PMS2+,c-erbB-2(GC) -,BRAF(-).

**Figure 3:**
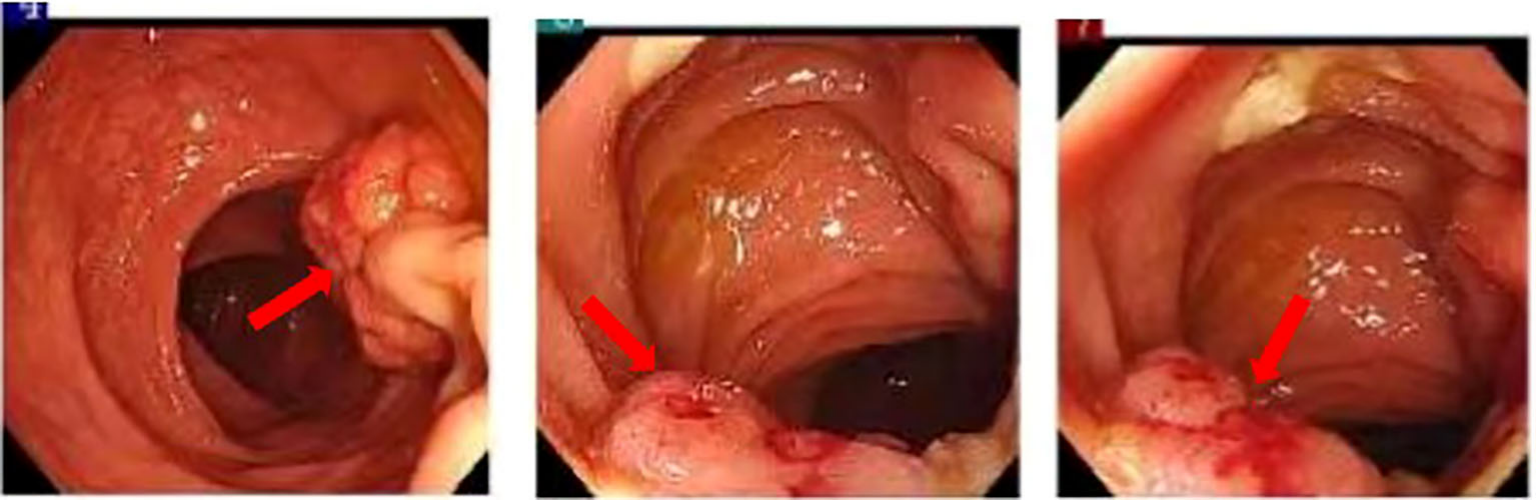
A semi-circumferential mass is observed at a distance of 20 cm from the anus, accompanied by localized intestinal wall torsion and slight mucosal stiffness.

Following comprehensive preoperative examinations, the patient underwent surgery for the tunica vaginalis. During the procedure, a mass was identified in the right testicular tunica vaginalis, prompting pathological examination of bilateral testicular tunica vaginalis and the mass. Pathological results indicated a potential digestive tract metastatic carcinoma(**Figure 2**). we proceeded to inquire further into the medical history. The patient reported indeed experiencing alterations in bowel habits, specifically noting a change in stool consistency characterized by thin and unformed stools. There were no instances of bloody stools, and the patient did not exhibit symptoms such as fever, weight loss, or anemia. Subsequently, a colonoscopy revealed a tumor located 20 cm from the anal verge, and tissue biopsy confirmed adenocarcinoma of the sigmoid colon(**Figure 3**). To assess abdominal infiltration and metastasis, an enhanced abdominal Computed Tomography scan demonstrated thickening of the colon wall, along with thickening of the peritoneum, omentum, and mesentery, accompanied by multiple soft tissue nodules, suggestive of metastasis.

**Figure 4:**
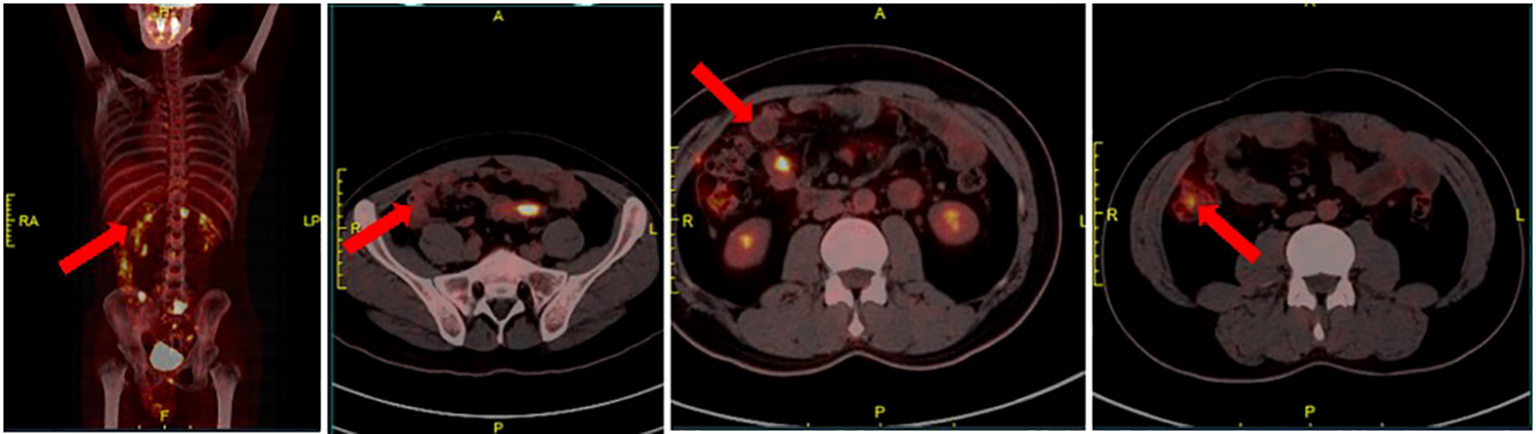
Local thickening of the transverse colon and sigmoid colon with concomitant elevated glucose metabolism, raising consideration for cancer; multiple soft tissue nodules with abnormal glucose metabolism in the peritoneal omentum, prompting consideration of metastatic carcinoma.

For a comprehensive evaluation of systemic multi-organ tissue metastasis and to guide further treatment strategies, the patient underwent a positron emission tomography scan. Results revealed localized lesions in the transverse and sigmoid colon, as well as multiple soft tissue nodules in the peritoneum and omentum(**Figure 4**). Following a detailed assessment of tumor metastasis, the hospital organized a multidisciplinary team (MDT) discussion, ultimately determining the treatment plan as XELOX (capecitabine and oxaliplatin) combined with bevacizumab.

After six months, the patient underwent eight cycles of neoadjuvant chemotherapy, and imaging indicated significant tumor reduction, meeting the criteria for tumor debulking surgery. However, a postoperative ultrasound revealed hydrocele in the right spermatic cord and tunica vaginalis. A collaborative procedure by the colorectal and urological surgery teams was performed to eliminate tumor cells. Intraoperatively, multiple miliary and sheet-like metastatic lesions were found at the pelvic floor, internal ring, right liver, spleen, greater omentum, and mesentery. The tunica vaginalis metastasis and the surface of the testis appeared normal. The principal surgeon performed radical resection of the sigmoid colon, excised intra-abdominal metastases, and completely removed the bilateral tunica vaginalis while preserving the testes.

Postoperatively, pathological examination confirmed moderately differentiated adenocarcinoma of the sigmoid colon(**Figure 2**), no special type, and immunohistochemistry revealed: P53 (+), ki67 (50%), MLH1 (present), PMS2 (present), MSH2 (present), MSH6 (present). These results were consistent with the previous findings in the testicular tunica vaginalis, confirming that the tunica vaginalis lesion was a metastasis from colon cancer. The patient received postoperative pain management, gastric protection, and other symptomatic treatments before discharge. Currently, one month postoperatively, the patient has commenced postoperative chemotherapy with the regimen of capecitabine and bevacizumab.

## Discussion

Differing from pediatric cases where hydrocele is often congenital, in adults, it is typically acquired. The most common form in adults is idiopathic hydrocele (8). Due to the extremely low incidence of testicular and peri-testicular cancers in the Western society, with 3 to 10 new cases per 100,000 males annually (9), hydrocele caused by neoplastic factors is rare. Research indicates that both primary and metastatic tumors can lead to hydrocele (10, 11), with a higher incidence observed in primary tumors. Rapidly developing hydroceles warrant consideration of malignancy possibilities (12), and ultrasound can be employed for initial assessment of testicular and peri-testicular masses. For some small lesions, as in the presented case, ultrasound sensitivity may be insufficient – preoperative ultrasound may not report abnormalities, necessitating further examinations such as Magnetic resonance imaging. The common approach to managing hydrocele is surgical intervention. Currently, there is some controversy regarding the necessity of pathological examination for routine patients (13). However, in the presented case, distinct proliferation was observed in the patient’s testicular tunica vaginalis during surgery, supporting the recommendation for routine pathological examination of testicular hydrocele with morphological abnormalities.

Testicular para-testicular tumors can be classified into primary and secondary types. Lipomas originating from the spermatic cord are the most common primary para-testicular tumors. Cystadenomas are the most common among epididymal tumors, followed by leiomyomas. In pediatric patients, embryonal carcinoma and rhabdomyosarcoma can be observed (14). Among primary testicular tunica tumors, the extremely rare malignant mesothelioma of the testicular tunica is noteworthy, with only a few hundred cases reported globally (15). The primary sites of origin for testicular and para-testicular metastatic cancers include the lungs, digestive tract, prostate, and kidneys. According to Charles W and colleagues, the most common primary tumors metastasizing to the testicles include prostate cancer (35%), lung cancer (18%), melanoma (11%), and renal cancer (9%) (16). The metastasis of digestive tract tumors to the testicles is overall rare. Li, Kulkarni, Seo, Liu, and others have reported cases of tumors from digestive organs such as the stomach, appendix, pancreas, and colon metastasizing to the testicles or para-testicular tissues (17–19). This often implies extensive peritoneal metastasis and is believed to be related to the anatomical structure of the human body, where the processus vaginalis provides a potential route for metastasis. It has been reported that the incidence of associated tunica fluid in testicular metastatic colon cancer is approximately 30%. Aydin and colleagues reported a case of childhood renal cell carcinoma metastasizing to para-testicular tissues, suspecting tumor spread directly from the abdomen along the processus vaginalis (20). Secondary tumors often present with multifocal lesions, significant growth in the testicular interstitium, and conspicuous involvement of blood vessels. This can be distinguished from primary tumors, and such differentiation holds guiding significance for subsequent treatment (12).

Through a literature search, six reports were identified regarding metastasis of colorectal cancer to the para-testicular tissues (**Table 1**). Colorectal cancer has the potential to metastasize to the testis, tunica vaginalis, epididymis, spermatic cord, and other sites. Common manifestations include involvement of the testis and surrounding tissues, with mucinous adenocarcinoma, characterized by a high malignant degree, being the predominant pathological type (2–7). In the presented case, intraoperatively, gross examination revealed involvement of the tunica vaginalis without evident signs of testicular metastasis.

**Table 1:** Six cases of metastasis from various types of colorectal tumors to the testes and adjacent tissues.

gender	age	primary tumor	metastasis site
male	NA (old man)	mucinous tumor of the cecum	the tunica vaginalis testis
male	53-year-old	colon adenocarcinoma	the right epididymal tail
male	62-year-old	colon mucinous adenocarcinoma	the spermatic cord
male	56-year-old	colon carcinoma	epididymis
male	65-year-old	colonic mucinous adenocarcinoma	testis, epididymis,spermatic cord
male	75-year-old	colonic mucinous cystadenocarcinoma	the tunica vaginalis testis

In China, the incidence and mortality rates of colorectal cancer are continuously increasing. The statistical results for 2020 indicate 555,000 new cases and 286,000 deaths (1). Colorectal cancer exhibits diverse pathological types, with adenocarcinoma being the most common (21). Other rare and specific types include mucinous adenocarcinoma, signet ring cell carcinoma, and medullary carcinoma, as reported by Liu et al., with two cases being mucinous adenocarcinoma. Histologically, the adenocarcinoma in this case belongs to the moderately differentiated type (G2) (22). Although histologically less malignant than mucinous adenocarcinoma, it still presented rare metastasis to the testicular region, likely due to advanced cancer staging and widespread metastasis. The expression analysis of DNA mismatch repair(MMR) proteins suggests proficient DNA mismatch repair (pMMR), and microsatelliteinstability(MSI) testing indicates microsatellite stability (MSS), indicating no indications for the use of PD-1/PD-L1 immune checkpoint inhibitors (23). The patient did not exhibit mutations in KRAS/NRAS/PIK3CA/BRAF, and there were no indications for the use of anti-EGFR drugs (such as cetuximab). Therefore, the xelox + bevacizumab regimen was adopted.

In the early stages of testicular and paratesticular tumors, apart from symptoms originating from the primary site, there is often no obvious discomfort in the scrotal region. Patients mostly rely on incidental discoveries during ultrasound examinations, with approximately 27% of testicular cancer patients presenting with scrotal swelling and pain (24). In advanced stages, patients typically exhibit scrotal enlargement, and physical examination may reveal hydrocele or scrotal masses. Takahiko Sakuma and colleagues reported a case of colon cancer metastasizing to the testicle, where preoperative ultrasound suggested testicular hydrocele, but pathological examination revealed testicular metastatic cancer. The tumor exhibited cystic changes, mimicking the presentation of testicular hydrocele (25). Therefore, for cases showing signs of hydrocele during physical examination, caution is needed, and relevant investigations should be diligently conducted.

Colorectal cancer can metastasize to the testicles and adjacent tissues through various mainstream pathways. These include: 1. Lymphatic spread from retroperitoneal lymph nodes or lymphatic vessels causing lymphatic obstruction, dilation, dysfunctional lymphatic valves leading to retrograde lymphatic extension, or retrograde venous spread; 2. Direct intraperitoneal dissemination along the inguinal canal, both internally and externally; 3. Hematogenous dissemination following the formation of arterial emboli (26); 4. Retrograde spread along the vas deferens (7). In analyzing the specific case, according to the research by Hassan et al.the short drainage distance of the sigmoid colon from the pre-aortic lymph nodes, where testicular lymph drainage also occurs, makes sigmoid colon tumors more prone to testicular metastasis (27).

However, in this case, the metastatic pathway may involve direct peritoneal dissemination through the Processus vaginalis. Before the second surgery for colon cancer, ultrasound revealed effusion in the spermatic cord and testicles. This is indicative of the presence of an unclosed inguinal canal, and the tumor likely migrated through the Processus vaginalis via the incompletely closed opening in the peritoneum, leading to spermatic cord sheath effusion. Why does the right and left differ,one theoretical explanation for this asymmetric progression could be the earlier descent of the left testicle from the peritoneum, with the later descent of the right testicle potentially allowing the tumor to spread through the residual vaginal membrane (28).

The patient with distant metastasis, exhibits a 5-year survival rate of approximately 15.6% according to SEER CANCER. Literature reports indicate a 3-year survival rate of about 23% (29). Without any intervention, the median survival time for patients with colorectal cancer accompanied by peritoneal metastasis is approximately 6 months (30, 31). For patients undergoing cytoreductive surgery and hyperthermic Intraperitoneal Chemotherapy, the Disease-Free Survival (DFS) is estimated to range between 9.8 and 12.6 months, with a median Overall Survival (mOS) of approximately 38.4 to 40.8 months (32). The therapy significantly prolongs the survival time of patients with relevant conditions. However, according to literature reports, the majority of colorectal cancer (CRC) patients experience recurrence following complete cytoreductive surgery (CRS) with hyperthermic intraperitoneal chemotherapy (HIPEC), with reported recurrence rates of approximately 70% during a 25-month follow-up (33). We still need to explore more effective treatment methods to address late-stage cancer patients.

## Conclusion

Although the metastasis of colorectal cancer to the testis is rare, it should not be overlooked in clinical practice. Clinicians should be aware of such uncommon metastases, and when a tumor patient presents with testicular hydrocele, recognize it as a potential manifestation of tumor metastasis.

## Data availability statement

The original contributions presented in the study are included in the article/supplementary material. Further inquiries can be directed to the corresponding author.

## Ethics statement

In the process of diagnosis and treatment, all examinations and procedures are conducted with the participant's informed consent, duly documented by signing an informed consent form. The participants provided their written informed consent toparticipate in this study. Written informed consent was obtained from the individual(s) for the publication of any potentially identifiable images or data included in this article.

## Author contributions

KX: Writing – original draft, Writing – review & editing, Conceptualization, Methodology. YZ: Investigation, Software, Writing – review & editing. MC: Investigation, Software, Writing – review & editing. ZL: Data curation, Supervision, Writing – review & editing. XH: Formal analysis, Funding acquisition, Project administration, Resources, Validation, Visualization, Writing – review & editing.
